# Supplementary material for: A burn-in(g) question: How long should an initial equal randomization stage be before Bayesian response-adaptive randomization?
Source: Stat Methods Med Res. 2026 Jan 19;35(11):2320–40. doi: 10.1177/09622802251411538 (PMC13744593; doi:10.1177/09622802251411538)
Supplement: Supplementary file 1 [file sj-pdf-1-smm-10.1177_09622802251411538.pdf]

Supplement to "A burn-in(g) question: How long should an initial equal randomization stage be before Bayesian response-adaptive randomization?"

1 Selection and description of BRAR trials with a burn-in period

Table 1: Characteristics of multiple response-adaptive clinical trials using a burn-in period. Reference (Ref.) number refers to the number in the bibliography list given below.

**Trial name/number(s)**	**Ref. number**	**Phase**	**Arms**	**Planned burn-in period**	**Target trial size**	**Early stopping**	**Justification of burn-in period**	**Randomization rule**	**Test(s) for primary endpoint**
BATTLE / NCT00409968, NCT00411671, NCT00411632, NCT00410059, NCT00410189	1	2	4	80	250	N	Lowest possible to ensure $\geq$ 1 patient in each marker group to complete treatment before starting RAR	BRAR with covariate adjustment	PPCS
BATTLE-2 / NCT01248247	2	2	4	70	200	N	Not specified	BRAR with covariate adjustment	None
ARREST / NCT03880565	3	2	2	30	$\leq$150	Y	Not specified	BRAR with clipping	PPCS (early stopping threshold 0.986)
ABT-089 / NCT00555204	4	2	7	$\geq$5 per arm	$\leq$400	Y	Not specified	BRAR	Posterior probability distributions/credible intervals
ADORE / NCT00807911	5	3	2	300	$\leq$1100	Y	Not specified	BRAR with variance adjustment	PPCS (early stopping threshold 0.99)
ESETT / NCT01960075	6	3	3	300	$\leq$795	Y	Not specified	BRAR	Posterior probability (interim, early stopping threshold 0.975), Chi-squared test (final)
PAIN-CONTRoLS/ NCT02260388	7	4	4	$\geq$20 per arm	$\leq$400	Y	Not specified	BRAR	Posterior probability (early stopping threshold 0.975)

Bibliography (RA clinical trial list)

1. Kim, E.S., Herbst, R. S., Wistuba, I. I., Lee, J. J., Blumenschein, G. R., Tsao, A., Stewart, D. J., Hicks, M. E., Erasmus Jr., J., Gupta, S., Alden, C. M., Liu, S., Tang, X., Khuri, F. R., Tran, H. T., Johnson, B. E., Heymach, J. V., Mao, L., Fossella, F., Kies, M. S., Papadimitrakopoulou, V., Davis, S. E., Lippman, S. M., Hong, W. K. (2011). *The BATTLE trial: Personalizing therapy for lung cancer. Cancer Discovery 1*(1), 44–53. https://doi.org/10.1158/2159-8274.CD-10-0010

2. Papadimitrakopoulou, V., Lee, J. J., Wistuba, I. I., Tsao, A. S., Fossella, F. V., Kalhor, N., Gupta, S., Byers, L. A., Izzo, J. G., Tang, X., Skoulidis, F., Gibbons, D. L., Shen, L., Wei, C., Diao, L., Peng, S. A., Wang, J., Tam, A. L., Heymach, J. V., Hong, W. K., Gettinger, S. N., Goldberg, S. B., Koo, J. S., Herbst, R. S., Miller, V. A., Coombes, K. R., Mauro, D. J., & Rubin, E. H. (2016). The BATTLE-2 study: A biomarker-integrated targeted therapy study in previously treated patients with advanced non-small-cell lung cancer. *Journal of Clinical Oncology, 34*(30), 3638–3647. https://doi.org/10.1200/JCO.2015.66.0084

3. D. Yannopoulos, J. Bartos, G. Raveendran, E. Walser, J. Connett, T. A. Murray, G. Collins, L. Zhang, R. Kalra, M. Kosmopoulos, R. John, A. Shaffer, R. J. Frascone, K. Wesley, M. Conterato, M. Biros, J. Tolar, and T. P. Aufderheide. Advanced reperfusion strategies for patients with out-of-hospital cardiac arrest and refractory ventricular fibrillation (ARREST): A phase 2, single centre, open-label, randomised controlled trial. *The Lancet, 396*(10265):1807–1816, 2020. https://doi.org/10.1016/S0140-6736(20)32338-2.

4. Lenz, R. A., Pritchett, Y. L., Berry, S. M., Llano, D. A., Han, S., Berry, D. A., Sadowsky, C. H., Abi-Saab, W. M., & Saltarelli, M. D. (2015). Adaptive, dose-finding phase 2 trial evaluating the safety and efficacy of ABT-089 in mild to moderate Alzheimer disease. *Alzheimer Disease & Associated Disorders, 29*(3), 192–199. https://doi.org/10.1097/WAD.0000000000000093

5. Carlson, S. E., Gajewski, B. J., Valentine, C. J., Kerling, E. H., Weiner, C. P., Cackovic, M., Buhimschi, C. S., Rogers, L. K., Sands, S. A., Brown, A. R., Mudaranthakam, D. P., Crawford, S. A., & DeFranco, E. A. (2021). Higher dose docosahexaenoic acid supplementation during pregnancy and early preterm birth: A randomised, double-blind, adaptive-design superiority trial. *EClinicalMedicine, 36*, 100905. https://doi.org/10.1016/j.eclinm.2021.100905

6. Chamberlain, J. M., Kapur, J., Shinnar, S., Elm, J., Holsti, M., Babcock, L., Rogers, A., Barsan, W., Cloyd, J., Lowenstein, D., Bleck, T. P., Conwit, R., Meinzer, C., Cock, H., Fountain, N. B., Underwood, E., Connor, J. T., Silbergleit, R., Neurological Emergencies Treatment Trials, & Pediatric Emergency Care Applied Research Network investigators. (2020). Efficacy of levetiracetam, fosphenytoin, and valproate for established status epilepticus by age group (ESETT): A double-blind, responsive-adaptive, randomised controlled trial. *The Lancet, 395*(10231), 1217–1224. https://doi.org/10.1016/S0140-6736(20)30611-5

7. Barohn, R. J., Gajewski, B., Pasnoor, M., Brown, A., Herbelin, L. L., Kimminau, K. S., Mudaranthakam, D. P., Jawdat, O., Dimachkie, M. M., & the PAIN-CONTRoLS Study Team. (2020). Patient Assisted Intervention for Neuropathy: Comparison of Treatment in Real Life Situations (PAIN-CONTRoLS): Bayesian adaptive comparative effectiveness randomized trial. *JAMA Neurology, 77*(5), 557–567. https://doi.org/10.1001/jamaneurol.2020.2590

2 Type I error rate and power results for the Wald test

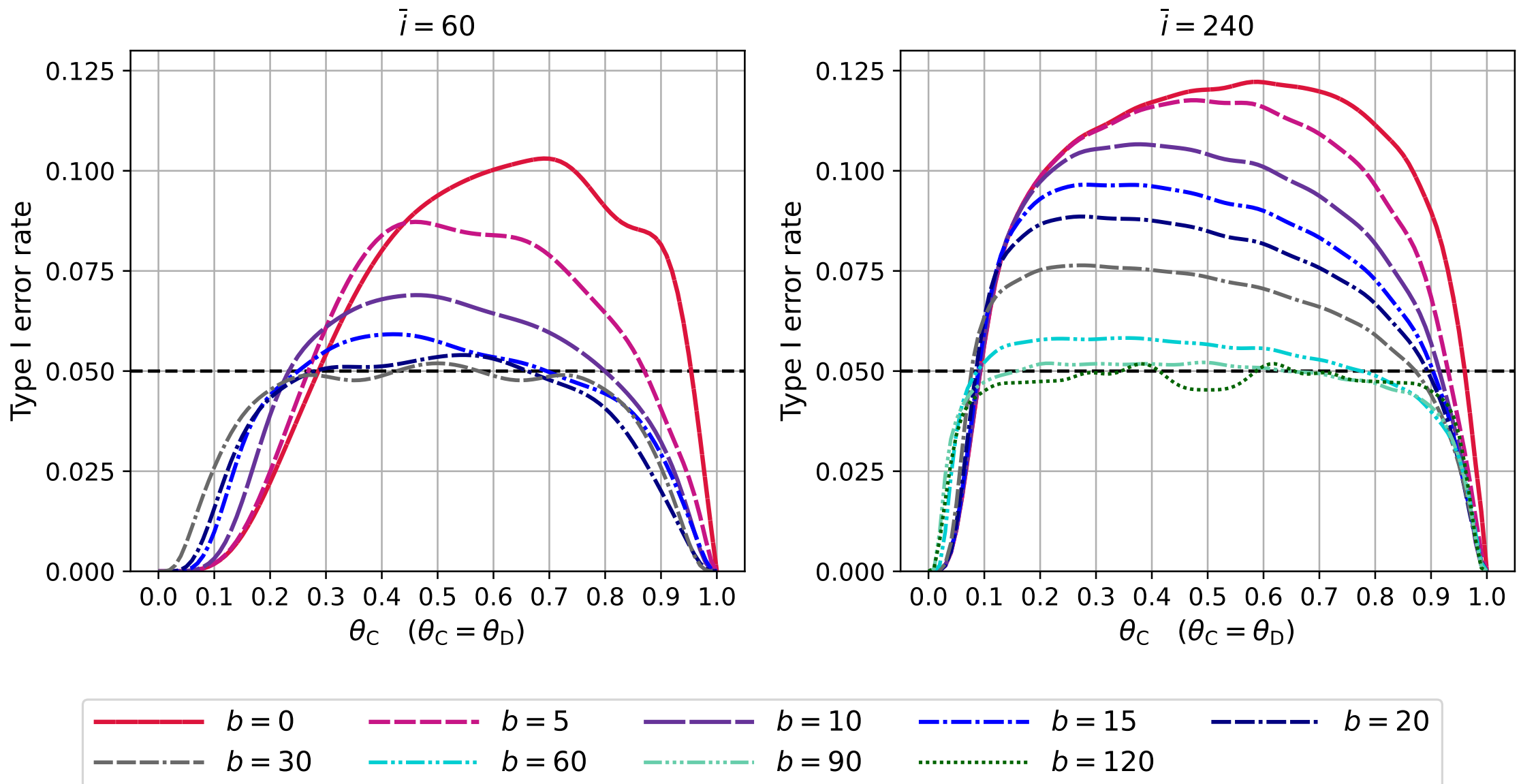

Figure 1: Type I error rate profiles for the Bayesian response-adaptive randomization design using the asymptotic Wald test for trial sizes $\bar{i} = 60$ and $\bar{i} = 240$, across $\theta_C = \theta_D \in \{0.00, 0.01, \ldots, 1.00\}$ for different values of burn-in length b. The significance level α was set to 0.05.

Table 2: Maximum type I error rate across the whole parameter space (using a grid approximation) (in %) of the asymptotic Wald test based on the posterior probability of control superiority (significance level $\alpha = 0.05$) under the Bayesian response-adaptive randomization design as burn-in varies. Burn-in proportion (BP) is the proportion of the trial within the burn-in stage, given by BP$= 2b/\bar{i}$. BP$= 1.0$ corresponds to equal allocation. Type I error rates above 6% are indicated in bold, with the severity of the inflation emphasized by a gradient (red being worst).

BP	0.00	0.10	0.20	0.30	0.40	0.50	0.60	0.70	0.80	0.90	1.00
$\bar{i} = 20$	**6.04**	**6.22**	**7.05**	**6.75**	**6.09**	5.34	4.76	5.90	5.53	5.12	4.22
$\bar{i} = 40$	**9.72**	**9.89**	**7.88**	**7.00**	**6.30**	**6.08**	5.65	5.94	5.69	5.16	5.19
$\bar{i} = 60$	**10.31**	**9.87**	**8.21**	**7.11**	**6.54**	5.92	5.52	5.16	5.17	5.20	5.19
$\bar{i} = 80$	**10.86**	**10.07**	**8.24**	**7.07**	**6.28**	5.81	5.47	5.32	5.09	5.08	5.67
$\bar{i} = 100$	**11.27**	**10.17**	**8.25**	**7.10**	**6.30**	5.82	5.57	5.29	5.23	5.22	5.69
$\bar{i} = 240$	**12.22**	**10.23**	**8.32**	**7.10**	**6.35**	5.83	5.54	5.26	5.11	5.10	5.18

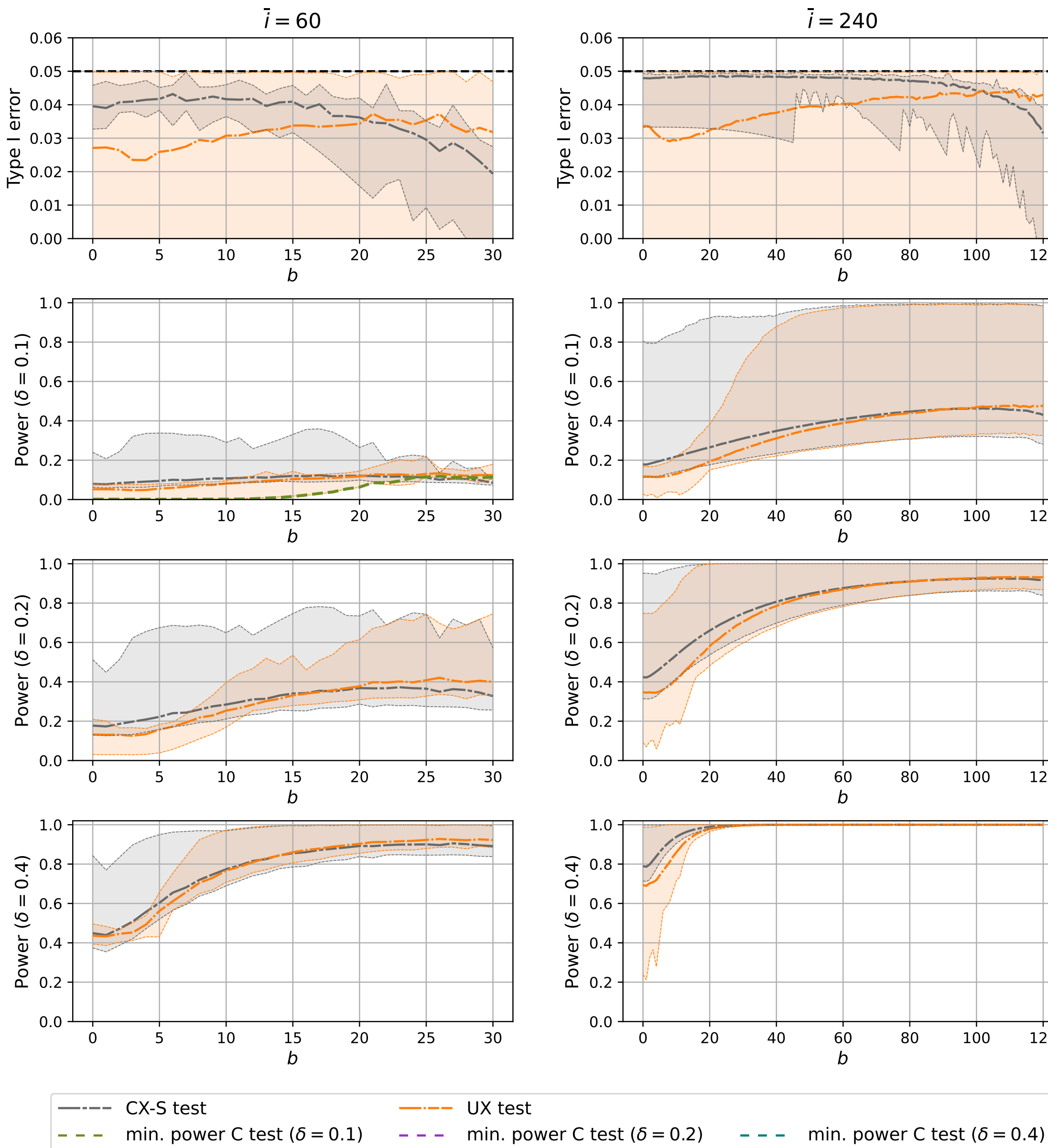

Figure 2: Type I error rate and power under treatment effects $\delta \in \{0.1, 0.2, 0.4\}$ for the Bayesian response-adaptive randomization design using the unconditional exact (UX) and conditional exact Wald test based on total successes (CX-S test) with trial size $\bar{i} = 60$ and $\bar{i} = 240$ across different burn-in lengths. For comparison we have plotted the (grid-approximated) minimum power for the asymptotic Wald test for $\bar{i} = 60$ and $\delta = 0.1$. The averages for each treatment effect δ and fixed burn-in length are represented by thick lines, while the minimum and maximum are presented by thin dotted lines and the ribbons. The significance level α was set to 0.05.

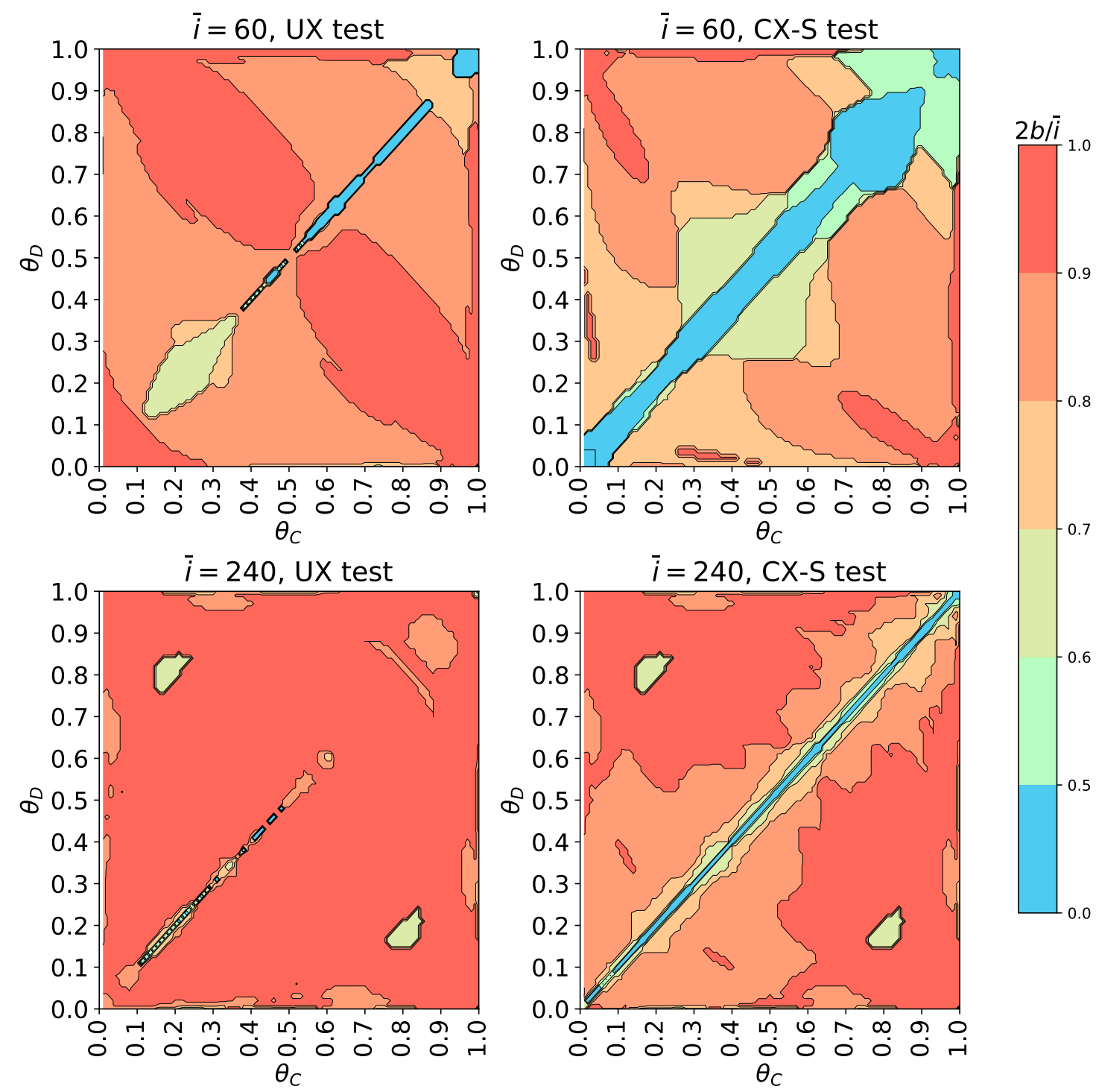

Figure 3: Optimal burn-in proportions $(2b/\bar{i})$ in terms of power of the Bayesian response-adaptive randomization design using the unconditional exact (UX) and conditional exact test based on total successes (CX-S test) based on the Wald test statistic for every parameter configuration in the grid $\theta_{\mathrm{C}}, \theta_{\mathrm{D}} \in \{0.00, 0.01, \ldots, 1.00\}$. The considered trial sizes are $\bar{i} = 60$ and $\bar{i} = 240$ participants.

3 Tables with critical values

Table 3: Upper critical values per burn-in length b for trial size $\bar{i} = 60$ for the calibration test based on the posterior probability of control superiority (PPCS), the unconditional exact (UX) test based on PPCS, conditional exact PPCS test based on total successes (CX-S PPCS test) for 12 and 48 total successes, as well as the upper critical values when using the Wald test statistic for these tests instead of the PPCS. The right significance level was set to 2.5%. Trailing zeros are not displayed.

b	Calibration (PPCS)	UX PPCS	CX-S PPCS (S=12)	CX-S PPCS (S=48)	UX Wald	CX-S Wald (S=12)	CX-S Wald (S=48)
0	0.9782333553957079	0.9947496072990083	0.9485264395008914	0.9927984157010034	2.302718174896149	1.6972660590567197	2.4972287576249435
1	0.9781225779090679	0.9953212845229524	0.9485264395008914	0.9927984157010034	2.302718174896149	1.6972660590567197	2.4972287576249435
2	0.9782512686272499	0.992067689053411	0.9485264395008914	0.9927984157010034	2.314198293489495	1.6972660590567197	2.4972287576249435
3	0.979026052548421	0.9916741508608418	0.9485264395008914	0.9861681852935872	2.3348799378810092	1.6972660590567197	2.056255801077067
4	0.9805617416069489	0.9916672873845505	0.9485264395008914	0.9861681852935872	2.3069908920018367	1.6972660590567197	2.056255801077067
5	0.9805617416069489	0.9898612626802341	0.9524953443194414	0.9861681852935872	2.244193077610112	1.6972660590567197	2.056255801077067
6	0.979483984434495	0.9884376484509019	0.957389825103261	0.9850237556333951	2.217970823170696	1.8390251085921658	2.019082882743081
7	0.979026052548421	0.9881797899514686	0.957389825103261	0.9850237556333951	2.1920662875450887	1.8571761720800164	2.019082882743081
8	0.9786845650880148	0.9861019884949895	0.957389825103261	0.9850237556333951	2.1496104640952374	1.8571761720800164	2.019082882743081
9	0.9786845650880148	0.9849109348027901	0.957389825103261	0.9794839844346975	2.146561442983005	1.8571761720800164	1.9302209789390594
10	0.9784545138738495	0.9847798890464465	0.9608623622030332	0.9848973252369821	2.1145598486192863	1.8571761720800164	2.0442480302927746
11	0.9782039937554963	0.9839067305268605	0.969962204991043	0.9794839844346975	2.0961796002305153	2.03874204282267	1.9302209789390594
12	0.9777091306875006	0.9839067305268605	0.969962204991043	0.9794839844346975	2.083022575693182	2.03874204282267	1.9048369267717764
13	0.97648627274697	0.9820200951877553	0.9768885222819681	0.9779145861440016	2.0660294406384994	2.2305545269340086	1.9302209789390594
14	0.97648627274697	0.9820200951877553	0.9663733717955911	0.9848973252369821	2.06568306450296	1.8390251085921658	2.03512144061183
15	0.976339153292231	0.9817933345684322	0.9608623622030332	0.9779145861440016	2.021702176265099	1.8390251085921658	1.9302209789390594
16	0.975982551964179	0.9800490065929534	0.9608623622030332	0.9775738544502989	2.021702176265099	1.8390251085921658	1.8905648266414632
17	0.9757257799772292	0.9797996486881216	0.9608623622030332	0.9779145861440016	2.0217021762650984	1.8390251085921658	1.9302209789390594
18	0.9754760637149139	0.9792993282725546	0.9707139671387696	0.9847392002468287	2.011594300028457	1.9827248472052368	2.0442480302927746
19	0.975982551964179	0.9775738544504023	0.9707139671387696	0.9779145861440016	2.011594300028457	1.9827248472052368	1.9302209789390594
20	0.9761502054406045	0.9775738544504023	0.9784545138739889	0.9779145861440016	2.011594300028457	2.108807359754724	1.9302209789390594
21	0.9749836110645047	0.9774557869401452	0.9726534336515967	0.9683933684856809	1.9639359620800905	1.9616525660360038	1.7936792983029057
22	0.9749836110645047	0.9754756964054718	0.9663733717955911	0.9830678759741764	1.9924984760531164	1.8199078886854556	1.9863639991535658
23	0.9749836110645047	0.9752350867338475	0.9663733717955911	0.9830678759741764	1.9924984760531164	1.8199078886854556	1.9863639991535658
24	0.9761854762466753	0.9766456353512373	0.9663733717955911	0.9830678759741764	2.0013843300632175	1.8199078886854556	1.9863639991535658
25	0.9742791605270222	0.9754760637149179	0.9726534336515967	0.9734846398942882	1.9638370552058695	1.9616525660360038	1.8627612616951987
26	0.9736258043787354	0.9741432202192297	0.9803217074512501	0.9631244804017428	1.9493917067918949	2.067785288547187	1.7377796424874383
27	0.9761854762466597	0.9761854762466597	0.9852683841251674	0.9515529995231619	1.9936203984193257	2.203560114236368	1.6059401548352457
28	0.9763391532922303	0.9769204877645723	0.9628077487730287	0.9845660747738446	2.010841003245581	1.7465500627584618	2.175406712823416
29	0.9785993142093378	0.9785993142093378	0.9628077487730287	0.9790555184650054	2.0455920583489493	1.7465500627584618	2.0074488153546177
30	0.9793538324121673	0.9793538324121673	0.9723027995475091	0.9723027995475023	2.06568306450296	1.8709439823564948	1.8709439823564944

Table 4: Upper critical values per burn-in length b for trial size $\bar{i} = 240$ for the calibration test based on the posterior probability of control superiority (PPCS), the unconditional exact (UX) test based on PPCS, conditional exact PPCS test based on total successes (CX-S PPCS test) for 48 and 192 total successes, as well as the upper critical values when using the Wald test statistic for these tests instead of the PPCS. The right significance level was set to 2.5%. Trailing zeros are not displayed.

b	Calibration (PPCS)	UX PPCS	CX-S PPCS (S=48)	CX-S PPCS (S=192)	UX Wald	CX-S Wald (S=48)	CX-S Wald (S=192)
0	0.9882676016349606	0.9978695860910114	0.9779237683765297	0.9935673601041999	2.4924269802206607	2.352504211231289	2.3591045036850353
3	0.9882781171698278	0.9966560452481168	0.9779237683765297	0.9935673601041999	2.4898635332677275	2.352504211231289	2.3081509468440293
6	0.9880079097443818	0.9949247272897915	0.9779237683765297	0.991084274255219	2.4685982065131458	2.352504211231289	2.172379970104011
9	0.9868496315212975	0.994149816164298	0.9789473370146099	0.9899618030235767	2.42347429432613	2.3674264179475606	2.1448807552941043
12	0.9859061873552727	0.9931069461247625	0.9795839919025272	0.9899618030235767	2.377308952207739	2.352504211231289	2.129651103799256
15	0.9848601466735132	0.9922694364958993	0.9795839919025272	0.9883302461014646	2.3342462996474693	2.290457193538588	2.0916576827580196
18	0.9840613752311802	0.991454930327456	0.9789473370146099	0.9875684653705998	2.297411946432368	2.281724872913192	2.0826766024115546
21	0.9834818358892811	0.99040429280052	0.9779237683765297	0.9863823116211614	2.2540856718479256	2.281724872913192	2.039125749132347
24	0.9825325957598087	0.989389294521634	0.9779237683765297	0.9851695377108294	2.225770355248036	2.2528422687314156	2.0299840102957667
27	0.982011361881802	0.9890253991729132	0.9779237683765297	0.9843824508623696	2.1972067198446776	2.1727101099763697	2.0102192304776025
30	0.9813789275654832	0.988098859326659	0.976712301838966	0.9841871719052863	2.173363798231878	2.167882408925361	2.001642162346673
33	0.9807354904452916	0.987111560547467	0.9759029039046823	0.9838671977559389	2.1505817024682106	2.151372384691242	2.001642162346673
36	0.9802003247812675	0.9860421383754918	0.9765353885456126	0.9837443758363629	2.1292461239382163	2.151372384691242	2.001642162346673
39	0.9798456572505423	0.9859446520122928	0.9759029039046823	0.9837443758363629	2.1152174018997143	2.120054830724824	2.001642162346673
42	0.9793251252552753	0.985801486990717	0.9759029039046823	0.9823065236195374	2.102010609415397	2.120054830724824	1.999388618240317
45	0.9789050751634131	0.9847863771303834	0.9759029039046823	0.9813391438152439	2.0831788130617026	2.095325560916048	1.9735885370565476
48	0.9782764539435482	0.9848259538928533	0.9757560212672464	0.9813391438152439	2.0717534025881643	2.095325560916048	1.9735885370565476
51	0.9780694041635337	0.9839935203884931	0.9757560212672464	0.9813391438152439	2.06247788425256	2.0838309093405654	1.9735885370565476
54	0.9777912401878406	0.9829776928095112	0.9757560212672464	0.979841597065226	2.054916109391418	2.0838309093405654	1.9622858889390855
57	0.97754615902655	0.9821405301601455	0.9729880772227908	0.979841597065226	2.0441817221674086	2.0111877063907055	1.9585508606473352
60	0.9771556810377969	0.9813949578663805	0.9729880772227908	0.979841597065226	2.0385605822215553	2.0111877063907055	1.9585508606473352
63	0.9770013457282741	0.9802356689696907	0.9729880772227908	0.9792028055195411	2.034639995935301	2.0111877063907055	1.9622858889390855
66	0.9767619635098954	0.9800704486354284	0.9729880772227908	0.9792028055195411	2.022816583553441	2.0111877063907055	1.9622858889390855
69	0.9764936364612985	0.9795346029830811	0.9745236227530435	0.9792028055195411	2.01261895935118	2.027380759777913	1.9622858889390855
72	0.9761389739573652	0.9789083257637078	0.9745236227530435	0.9773763117006349	2.0091250451709657	2.027380759777913	1.934637329247149
75	0.9760426594920191	0.9803586199033842	0.9722155756458432	0.9773763117006349	2.0014074321358986	1.9732174321385578	1.934637329247149
78	0.9757415734427812	0.9798372471904924	0.9744379445372018	0.9793750452494494	1.9996422180924787	1.9989414188733639	1.9764062307123254
81	0.9757123634191115	0.9783026447072881	0.9744379445372018	0.9780120571464848	1.9996422180924787	1.9989414188733639	1.957865318832861
84	0.9755251334871501	0.9774463867226041	0.9744379445372018	0.9780120571464848	1.9958109889528413	1.9989414188733639	1.957865318832861
87	0.9753831265757733	0.9773137969705136	0.9729055089671458	0.9766902783228439	1.9846900071524582	1.9645630931448923	1.9448083085077992
90	0.9753200810730491	0.9783167736432662	0.9766494332233285	0.9755905293485424	1.981391894823088	2.0322910658090607	1.928933952096659
93	0.9753473851845181	0.976715886004717	0.9716880143896327	0.9755905293485424	1.979380805888517	1.9328151013695574	1.928933952096659
96	0.9751577527104112	0.976958866579135	0.9746109508557322	0.9779137701086565	1.979380805888517	1.9752558436969372	1.9807451189307403
99	0.9750008632856821	0.9761930814934972	0.9746109508557322	0.9748917874938483	1.9812003226878416	1.9752558436969372	1.920685659421043
102	0.9750514703679901	0.9759518498508506	0.9738391142340772	0.9779137701086565	1.970179692688237	1.9563613920693081	1.9807451189307403
105	0.9750514703680765	0.9756342185057608	0.9738391142340772	0.9739593959105642	1.9702203519553598	1.9563613920693081	1.9153686624654291
108	0.9747306272212816	0.975320081067102	0.9733655062190011	0.9772698848404712	1.970246478514981	1.9403654227170288	1.9758440707257043
111	0.9747306272212816	0.9760038975299541	0.977083942124909	0.9691609033522085	1.9663602208709365	2.004543428664563	1.8495154916631973
114	0.9745692173455381	0.9754534449717089	0.9730326441004481	0.9769231392163866	1.9710902033222655	1.92605004476622	1.977535155785793
117	0.9741029707535324	0.9754768994775589	0.9802003247807616	0.9767689265551889	1.9736254009318919	2.056201246175504	1.9832169247701181
120	0.9734159553522349	0.9753797363964998	0.9729459236386523	0.9729459236390567	1.9711384650967143	1.9196434428611089	1.919643442861108

4 Additional results ARREST trial analysis

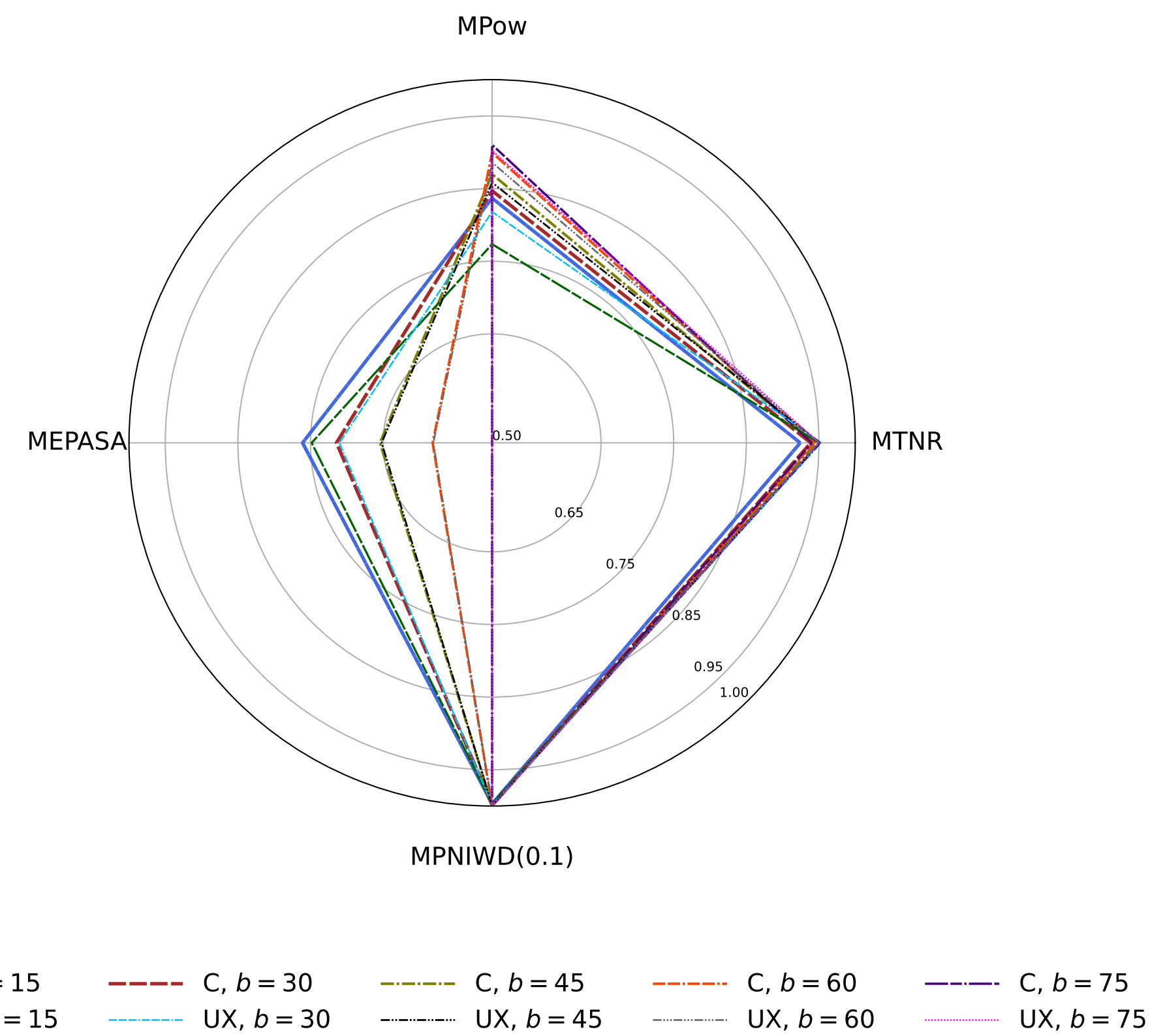

Figure 4: Star plot comparing the minimum expected proportion of allocations on the superior arm (MEPASA), minimum power (MPow), minimum true negative rate over interval $\theta_{\mathrm{C}} = \theta_{\mathrm{D}} \in \{0.02, 0.03, \ldots, 0.22\}$ (MTNR), and minimum probability of no imbalance in the wrong direction (MPNIWD(0.1)) for the ARREST trial when using a calibrated (C) and unconditional exact (UX) optional stopping threshold combined with a burn-in b of 15, 30, 45, 60, and 75 participants per arm. The minimum is taken over $\theta_{\mathrm{C}} = \theta_{\mathrm{D}} - \delta \in \{0.02, 0.03, \ldots, 0.22\}$ where $\delta = 0$ for the null hypothesis and $\delta = 0.25$ for the alternative hypothesis.

5 Tables for sensitivity analysis with respect to prior

Table 5: Average and maximum type I error rates (percentage points) under Bayesian response-adaptive randomization for different prior configurations, trial sizes $\bar{i}$, and burn-in lengths b. Type I error rate inflation above the 6% level is indicated by bold (and a color gradient from brown to red).

		$\bar{i}=60$						$\bar{i}=240$					
		AFP-C	MFP-C	AFP-CX	AFP-UX	AFP-W	MFP-W	AFP-C	MFP-C	AFP-CX	AFP-UX	AFP-W	MFP-W
Prior	b												
Beta(1.0, 1.0)	0	5.59	**14.53**	4.00	1.02	**6.38**	**10.31**	5.06	**13.31**	4.75	0.77	**9.56**	**12.22**
	$\bar{i}/10$	4.34	**8.59**	4.17	2.25	5.08	**8.21**	4.61	**7.82**	4.85	2.67	**6.45**	**8.32**
	$\bar{i}/4$	4.18	**6.36**	4.02	3.10	4.04	5.92	4.70	**6.30**	4.81	3.75	4.88	5.83
	$\bar{i}\cdot 4/10$	4.02	5.15	3.15	3.91	3.78	5.17	4.67	5.17	4.53	4.32	4.51	5.11
	$\bar{i}/2$	3.36	4.69	1.94	3.36	3.88	5.19	4.98	5.66	3.15	4.43	4.41	5.18
Beta(.01, .01)	0	3.13	4.97	3.18	3.13	4.22	**9.16**	**13.83**	**60.65**	4.55	1.72	**7.82**	**12.30**
	$\bar{i}/10$	**18.18**	**72.28**	3.73	1.18	4.66	**8.22**	**9.03**	**73.92**	4.76	0.43	**6.28**	**8.41**
	$\bar{i}/4$	**12.65**	**68.68**	3.77	0.96	3.94	5.91	**7.31**	**68.13**	4.71	0.28	4.86	5.82
	$\bar{i}\cdot 4/10$	**10.51**	**56.41**	3.02	0.90	3.80	5.17	**6.63**	**51.99**	4.44	0.35	4.51	5.12
	$\bar{i}/2$	**9.93**	**49.59**	1.94	0.80	3.88	5.19	**6.92**	**42.05**	3.15	0.34	4.41	5.18
Beta(0.5, 0.5)	0	4.63	**10.15**	3.63	1.61	**6.17**	**11.28**	4.01	**6.84**	4.56	2.17	**9.99**	**13.63**
	$\bar{i}/10$	3.87	5.73	4.10	3.28	4.95	**8.51**	4.78	**6.11**	4.82	3.82	**6.40**	**8.41**
	$\bar{i}/4$	4.52	**7.14**	3.89	3.16	4.00	5.92	5.01	**7.26**	4.77	3.51	4.87	5.83
	$\bar{i}\cdot 4/10$	4.56	5.81	3.02	3.69	3.80	5.17	4.94	5.69	4.52	3.18	4.51	5.12
	$\bar{i}/2$	4.18	5.53	1.94	3.95	3.88	5.19	5.32	**6.62**	3.15	4.60	4.41	5.18
Beta(1.4, 0.6)	0	5.30	**14.34**	4.30	1.29	**6.18**	**9.34**	4.72	**11.03**	4.87	1.32	**8.65**	**10.89**
	$\bar{i}/10$	4.54	**9.95**	4.36	1.90	5.17	**8.05**	4.43	**7.59**	4.88	2.70	**6.49**	**8.22**
	$\bar{i}/4$	4.33	**7.99**	4.14	2.36	4.07	5.94	4.62	**7.73**	4.81	3.05	4.90	5.84
	$\bar{i}\cdot 4/10$	3.92	5.52	3.11	3.26	3.77	5.17	4.67	5.87	4.55	3.28	4.51	5.12
	$\bar{i}/2$	3.57	4.69	1.94	3.57	3.88	5.19	5.05	**6.62**	3.15	4.50	4.41	5.18
Beta(0.6, 1.4)	0	**7.05**	**34.44**	3.36	0.81	5.60	**10.11**	**6.14**	**37.68**	4.16	0.44	**10.60**	**14.41**
	$\bar{i}/10$	3.93	**6.87**	3.91	2.64	4.86	**8.24**	4.91	**8.21**	4.82	2.69	**6.33**	**8.34**
	$\bar{i}/4$	4.32	5.72	3.86	3.80	3.99	5.90	4.87	5.96	4.80	4.02	4.86	5.82
	$\bar{i}\cdot 4/10$	4.18	4.99	3.18	4.12	3.79	5.18	4.80	5.11	4.52	4.70	4.51	5.12
	$\bar{i}/2$	3.57	4.69	1.94	3.57	3.88	5.19	5.05	**6.62**	3.15	4.50	4.41	5.18

AFP-C: average type I error (false positive) rate for the calibrated posterior probability of control superiority (PPCS) test; MFP-C: maximum type I error (false positive) rate for the calibrated PPCS test; AFP-CX: average type I error (false positive) rate for the conditional exact PPCS test; AFP-UX: average type I error (false positive) rate for the unconditional exact PPCS test; AFP-W: average type I error (false positive) rate for the asymptotic Wald test; MFP-W: maximum type I error (false positive) rate for the asymptotic Wald test. Beta(α, β) denotes the beta distribution with parameters α, β.

Table 6: Average operating characteristics (percentage points) under Bayesian response-adaptive randomization for different prior configurations, trial sizes $\bar{i}$, treatment effects δ, and burn-in lengths b. Changes in OCs for each non-uniform prior with the uniform (Beta(1.0, 1.0)) prior are displayed, with positive changes overlined (orange) and negative changes underlined (blue).

			$\bar{i}=60$						$\bar{i}=240$					
Prior	δ	b	MP-C	MP-CX	MP-UX	MP-W	EPASA	Bias	MP-C	MP-CX	MP-UX	MP-W	EPASA	Bias
Beta(1.0, 1.0)	0.10	0	0.18	6.23	0.00	0.51	65.56	3.78	4.22	11.37	0.10	28.33	78.35	4.58
		$\bar{i}/10$	0.16	6.89	0.03	0.55	64.82	2.09	18.98	18.96	6.06	26.84	76.43	1.70
		$\bar{i}\cdot 4/10$	11.10	9.10	7.90	11.86	54.72	0.10	33.02	32.04	32.14	33.37	57.85	0.06
	0.20	0	3.98	13.05	0.12	13.64	76.72	6.26	30.71	31.42	1.07	53.49	89.86	6.55
		$\bar{i}/10$	4.61	16.63	0.94	16.37	75.19	3.20	58.52	57.64	46.30	68.73	86.12	1.44
		$\bar{i}\cdot 4/10$	31.89	28.03	31.07	33.63	57.67	0.11	86.51	85.85	85.97	86.69	59.75	0.01
	0.40	0	39.65	37.44	4.16	61.39	88.61	7.57	79.86	70.93	10.23	76.24	96.21	7.20
		$\bar{i}/10$	61.39	55.97	36.48	70.66	85.61	2.73	99.08	98.89	97.87	99.48	89.80	0.21
		$\bar{i}\cdot 4/10$	87.20	84.60	86.85	88.33	59.78	0.03	100.00	100.00	100.00	100.00	59.94	0.00
Beta(.01, .01)	0.10	0	2.72	2.49	0.07	0.25	65.07	3.40	10.94	7.59	0.76	7.84	76.13	3.85
		$\bar{i}/10$	7.61	5.21	0.05	0.18	65.82	2.14	18.66	18.50	0.61	26.75	76.73	1.72
		$\bar{i}\cdot 4/10$	11.08	8.95	0.66	11.84	54.94	0.10	33.05	32.03	4.16	33.32	57.89	0.06
	0.20	0	5.60	5.40	1.77	3.66	75.27	5.60	30.87	22.71	4.54	30.16	87.55	5.39
		$\bar{i}/10$	17.31	13.24	0.97	4.25	76.15	3.19	56.55	56.71	5.66	68.49	86.23	1.43
		$\bar{i}\cdot 4/10$	31.83	27.75	4.18	33.61	57.82	0.11	86.51	85.86	42.01	86.66	59.79	0.01
	0.40	0	17.65	11.64	13.78	20.32	87.07	6.69	67.80	66.67	16.04	64.47	94.82	4.39
		$\bar{i}/10$	54.77	49.08	7.73	68.39	85.95	2.36	98.70	98.84	64.54	99.51	89.69	0.16
		$\bar{i}\cdot 4/10$	87.16	84.39	43.78	88.30	59.77	0.02	100.00	100.00	99.87	100.00	59.99	0.00
Beta(0.5, 0.5)	0.10	0	0.54	5.37	0.03	0.25	66.14	4.28	5.47	9.86	1.32	15.81	78.48	4.88
		$\bar{i}/10$	0.53	5.84	0.04	0.24	65.39	2.22	19.11	18.72	16.38	26.82	76.64	1.73
		$\bar{i}\cdot 4/10$	11.09	9.03	7.15	11.85	54.83	0.10	33.06	32.04	26.69	33.33	57.88	0.06
	0.20	0	6.12	11.21	1.29	5.58	77.42	7.10	25.19	26.86	5.47	51.78	90.01	7.10
		$\bar{i}/10$	7.44	14.48	2.20	7.19	75.84	3.38	57.76	57.21	52.84	68.64	86.23	1.45
		$\bar{i}\cdot 4/10$	31.86	27.89	29.58	33.62	57.76	0.11	86.52	85.87	82.10	86.67	59.80	0.01
	0.40	0	37.87	31.16	12.11	45.99	89.27	8.67	70.16	64.84	17.68	79.41	96.37	7.87
		$\bar{i}/10$	57.43	54.19	52.21	70.10	86.02	2.80	98.92	98.87	98.22	99.49	89.81	0.20
		$\bar{i}\cdot 4/10$	87.18	84.51	85.78	88.32	59.79	0.03	100.00	100.00	100.00	100.00	60.00	0.00
Beta(1.4, 0.6)	0.10	0	0.24	7.34	0.01	0.96	64.93	3.21	6.31	13.66	0.42	25.20	77.77	3.81
		$\bar{i}/10$	0.19	7.67	0.02	1.02	64.23	1.96	15.40	19.49	5.36	27.48	75.94	1.66
		$\bar{i}\cdot 4/10$	6.37	9.12	6.01	11.86	54.69	0.09	33.09	32.06	27.72	33.34	57.84	0.06
	0.20	0	5.93	15.82	0.35	23.80	75.68	5.28	42.38	39.34	5.56	50.93	89.17	5.44
		$\bar{i}/10$	4.57	18.37	0.80	26.06	74.35	3.04	59.79	58.90	48.49	70.09	85.82	1.47
		$\bar{i}\cdot 4/10$	32.06	28.06	28.07	33.63	57.64	0.10	86.57	85.89	82.89	86.68	59.79	0.01
	0.40	0	49.96	46.76	9.59	60.45	87.47	6.47	86.96	81.57	42.77	79.93	95.72	6.05
		$\bar{i}/10$	62.87	59.77	27.37	72.78	85.00	2.73	99.16	98.98	98.18	99.58	89.77	0.23
		$\bar{i}\cdot 4/10$	87.67	84.63	84.86	88.33	59.77	0.03	100.00	100.00	100.00	100.00	60.00	0.00
Beta(0.6, 1.4)	0.10	0	0.19	5.03	0.00	0.28	65.80	4.10	2.97	8.61	0.03	20.45	78.39	5.51
		$\bar{i}/10$	0.18	5.81	0.05	0.27	65.46	2.20	18.54	18.45	10.98	26.19	76.89	1.72
		$\bar{i}\cdot 4/10$	11.73	9.02	7.30	11.85	54.76	0.10	33.05	32.03	32.63	33.32	57.88	0.06
	0.20	0	3.43	10.63	0.06	6.25	77.33	6.95	15.45	23.61	0.32	52.45	90.21	7.99
		$\bar{i}/10$	4.01	14.37	1.64	8.05	76.04	3.34	57.17	56.28	43.86	67.25	86.41	1.41
		$\bar{i}\cdot 4/10$	33.11	27.84	31.73	33.63	57.71	0.11	86.52	85.87	86.26	86.67	59.79	0.01
	0.40	0	29.19	29.37	2.17	43.28	89.58	8.20	63.83	47.62	1.81	68.89	96.65	8.56
		$\bar{i}/10$	57.91	53.26	48.79	68.30	86.18	2.69	98.99	98.71	97.44	99.36	89.83	0.19
		$\bar{i}\cdot 4/10$	87.92	84.48	87.11	88.33	59.78	0.03	100.00	100.00	100.00	100.00	60.00	0.00

MP-C: minimum power of the calibrated posterior probability of control superiority (PPCS) test; MP-CX: minimum power of the conditional exact PPCS test; MP-UX: minimum power of the unconditional exact PPCS test; MP-W: minimum power of the asymptotic Wald test; EPASA: average expected proportion of allocations on the superior arm.
